# Use of Hormones Among Trans Women in the West Coast of Peninsular Malaysia: A Mixed Methods Study

**DOI:** 10.1089/trgh.2020.0119

**Published:** 2022-06-13

**Authors:** Abdul Rashid, Siti Nur Afiqah, Yufu Iguchi

**Affiliations:** ^1^ Department of Public Health Medicine, RCSI & UCD Malaysia Campus, George Town, Malaysia.; ^2^ Centre for Research on Women and Gender (KANITA), University Sains Malaysia, George Town, Malaysia.; ^3^ College of Asia Pacific Studies, Ritsumeikan Asia Pacific University, Beppu, Japan.

**Keywords:** trans women, hormone, Malaysia, estrogen, progesterone

## Abstract

**Purpose:**

There are no national data on hormone use by trans women in Malaysia. The objective of this study was to determine hormone use and the associated factors by trans women in Malaysia.

**Methods:**

This mixed method (quantitative and qualitative) study (JPEC 03-18-0021) was conducted using a self-administered questionnaire among trans women who used hormones and recruited using snow ball sampling method. All participants had undergone a blood test in an assigned laboratory. Besides total testosterone and total estradiol blood levels, renal and liver function tests and lipid profile were done.

**Results:**

A total of 111 out of the 141 respondents who participated were taking hormones. The main reason for not taking hormones was the concern for side effects. The main source of information concerning hormones was friends, and most procured the hormones from pharmacy without prescription and without first undergoing a physical or blood examination. All were on estrogens and only about half were on progesterone. The common mode of intake was oral and by injection. Most were on <4 mg of estrogens and did not report any major complication. Most rated the hormone affordability and satisfaction as good. Most had inadequate testosterone and estradiol blood levels. Except for total cholesterol and low-density lipoproteins, all other blood tests were normal. Correlation between serum testosterone and estradiol (*R*^2^ 0.012. B−9.273 (95% confidence interval −16.44 to −2.11). *p*=0.012) was statistically significant.

**Conclusion:**

The prevalence of hormone use was high, mostly nonprescription use and with no medical supervision.

## Introduction

Transgender is an umbrella term for people whose gender identity does not match their assigned sex at birth.^1,2^ Trans men are men who were assigned female at birth, trans women are women who were assigned male at birth, and cisgender refers to people whose gender identity matches their sex assigned at birth.^1,3^ Trans people who experience discontent with their assigned gender may experience gender incongruence,^4^ or gender dysphoria, an experience of profound psychological distress caused by the mismatch of gender identity and assigned sex.^5–7^

Hormonal therapy has been shown to improve mental health and improve quality of life in people with gender dysphoria.^1,8^ Hormonal treatment can induce changes for the perceived gender when properly prescribed,^8^ by a doctor trained in hormonal use among trans people. An effective and safe hormonal regimen that is personalized^2,8,9^ can suppress endogenous sex hormone secretions and maintain sex hormones at levels within normal range of the individual's affirmed gender^3^ after a confirmed diagnosis of gender dysphoria/incongruence and after clients are informed of the possible risks and benefits.^3^

Estrogen is the cornerstone for feminization of trans women.^6,8,10^ Antiandrogens are used to lower the testosterone levels and enhance effects of estrogens.^3,10–12^ The goal is to achieve feminization with mammary gland growth, body fat redistribution, decreased muscle mass, changes in hair growth, and in sexual function and mood improvement.^8^ The recommended regimens include oral estradiol 2–6 mg/day, transdermal patch 0.0025–0.2 mg/day, parenteral estradiol valerate or cypionate 5–30 mg intramuscular every 2 weeks or 2–10 mg every week. Antiandrogens recommended include spironolactone 100–300 mg/day, cyproterone acetate 25–50 mg/day or gonadotrophin-releasing hormone 3.75 mg monthly or 11.25 mg 3 monthly.^3,8^

Taking single or multiple hormones to increase the dose or different routes of administration do not necessarily quicken changes.^8^ Pills are convenient but not recommended for smokers, patches are safe but must be worn at all times, and injections cause very high and fluctuating estrogen levels.

Hormone therapy for adult trans women is relatively safe.^13^ However, estrogen blood level should be measured to ensure that endogenous sex steroids are suppressed and the administered sex steroids are maintained in the normal physiological range^3,8,10,14^ and risk for thromboembolism mitigated.^1,10^

Different cultures have varied levels of tolerance to trans people, which may influence the transition process.^3^ In the United Kingdom, Netherlands, United States, and Iran, transgender care is better accessed^15,16^ compared with most traditional patriarchal Muslim societies.^17^In Malaysia, the traditional classification of sex is male and female and is deeply ingrained in culture, law, and policies. This may have contributed to the discrimination of the trans community.^18,19^ Malaysia criminalizes transgender practices.^20^

In Malaysia, the judiciary comprises both civil and Sharia courts, which have jurisdiction over Islamic family law matters.^21^ The Sharia criminal enactment includes the provision of criminalizing a man posing as a woman. There are reports that federal and civil police have at times arrested trans women under the provision of the secular federal criminal code that prohibits public indecency. In addition, Muslim trans women are also bound by a fatwa (a nonbinding Islamic decree) issued in 1982 by the National Fatwa Council prohibiting Muslims from undergoing sex reassignment surgery, which resulted in the National Registration Department refusing trans people to change the gender for any reason.^20^

In Malaysia, hormones can only be prescribed and sold by registered medical practitioners or licensed pharmacists. Public hospitals do not provide hormones for trans persons. Most trans people buy hormones from drug stores or from across the border after consulting friends or the internet. This nonprescription hormone use may endanger health,^22,23^ hence the objective of this study was to determine hormone use by trans women in Malaysia and the factors associated with this use. The information garnered from this study can be used to improve the health of trans women in Malaysia.

## Methods

### Study design

This quantitative (cross-sectional) and qualitative study was conducted from 2019 to 2020.

#### Population and sampling

Nongovernmental organizations (NGOs) working with trans communities are usually registered under the guise of other activities. One such NGO that advocates and helps trans women with ∼300 members in west coast of Malaysia agreed to help the investigators conduct this study. Owing to the sensitive nature of the study, participants who were or had been in contact with the NGO were recruited using snowball sampling method with the help of the NGO personnel, who passed the questionnaire with a participant information sheet to consenting trans women. Once completed, the questionnaires were returned back to the researchers through the NGO.

Using EpiInfo software to calculate sample size, 105 participants were required based on 88% anticipated prevalence of hormone use^24^ in 300 trans women with level of significance of 5% and precision value of ±0.05. In total, 150 trans women were approached and questionnaire handed out with the anticipation of refusal to participate or rejection of incomplete questionnaires. For the qualitative part of the study, the NGOs were asked to identify trans women who were talkative in nature and were willing to be interviewed face-to-face. The investigators were cognizant of the risk of bias in collecting qualitative data. They did not influence but encouraged the participants to provide honest answers and to freely express their views.

#### Tool

A self-administered questionnaire used was adapted from a study by Sanchez et al.^25^ Questions related to hormone use were adopted and questions related to hormone types and place procured were added based on the suggestions given by the NGO. An NGO member, who is also a trans woman, was recruited and trained as a research assistant. Questions for the quantitative component of the study included demographics and details relating to hormone intake. Estrogens and antiandrogens were identified and recorded by the researchers from the medications that the participants stated they were taking, which included oral and injectable contraceptives and other sources.

For the qualitative part, in-depth interviews were conducted using a semistructured interview guide in the Malay language until saturation of information was achieved. The interviews focused on the reasons for taking hormones, source, satisfaction, unmet needs, and side effects. Only participants who completed the questionnaire were given a voucher to conduct blood tests in an assigned laboratory.

Total testosterone level of <55 ng/dL and estradiol level between 100 and 200 pg/dL were considered adequate feminization levels.^3,26^ Other blood tests, including lipid profile, renal and liver function tests were conducted to monitor for possible adverse effects of hormone use.^8^ Because of the inconsistent use of hormones by the participants leading to the possibility of androgen activity in the retained gonads, the normal value range for men provided by the laboratory were used; urea (1.7–8.4 mmol/L), creatinine (63.6–110.5 μmol/L), potassium (3.5–5.1 mmol/L), total cholesterol (up to 5.2 mmol/L), triglycerides (<1.7 mmol/L), low-density lipoproteins (LDL; <2.6 mmol/L), total bilirubin (2.0–25 μmol/L), alkaline phosphatase (40–150 U/L), serum glutamic pyruvic transaminase (0.0–55 U/L), and serum glutamic oxaloacetic transaminase (5.0–34 U/L).

The results of the blood test were given to the participants with notes concerning the results through the NGO representative, and they were told to contact the investigators if they needed clarification. Those with abnormal results were advised to consult their personal physicians.

#### Analysis

Data were analyzed using SPSS version 18 and Stata SE13. Prevalence of hormonal use and factors associated with the use are reported. Correlation of total estrogen and testosterone is presented. The qualitative data were recorded and transcribed verbatim in Malay, organized, coded, and recoded using N vivo version 12 by one of the investigators. The clustered codes were elicited as common themes by the research team.

#### Ethics

Ethical approval was received from Joint Penang independent Ethical Committee (JPEC 03-18-0021). After reading the information sheet, which stated the reasons, benefits, and their rights not to participate or to withdraw from the study at any time, they were asked to give a written informed consent. Each participant was given a unique identifier code to ensure confidentiality. The data are only accessible to the principal investigator.

## Results

The response rate was 94%, 141 of the 150 trans women approached responded.

The mean age was 33 years (18–54). Most were single (*n*=138, 97.9%), highest level of education up to secondary school (*n*=88, 62.4%), working full time (*n*=107, 75.9%), a median income of RM2000 (USD1=RM4), and living alone (*n*=56, 39.7%) or with friends (*n*=35, 24.8%). In total 13 in-depth interviews were conducted, the mean age of the respondents was 37 years, most were single (*n*=12, 92.3%), highest level of education up to secondary school (*n*=8, 61.5%), working full time (*n*=9, 69.2%), and living alone (*n*=5, 38.5%) or with friends (*n*=5, 38.5%). Of the 141 respondents, 111 (81.6%) were taking hormones. Their mean age was 33.2 years (17–51). Most were single (*n*=108, 97.3%), highest level of education up to secondary school (*n*=66, 59.5%), in full-time occupation (*n*=85, 76.6%) and staying on their own (*n*=43, 387%).

**FIG. 1.**
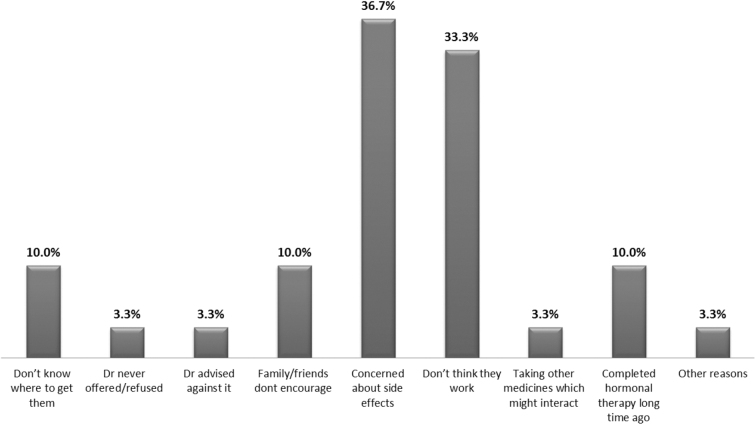
Reasons for not taking hormones.

Figure 1 shows the main (36.7%) reason for not taking hormones was the concern for side effects.

Table 1 shows the variables related to hormone use among the 111 respondents. Majority (87.4%) reported that friends were the main source of information concerning hormonal use and most (71.2%) procured the hormones from pharmacy without prescription.

**Table 1.** Hormonal Use and Associated Factors

Variable	*N*	%
Are you presently taking any hormones for gender realignment?		
Yes	115	81.6
No	26	18.4
Source of information concerning hormonal therapy (multiple choice)		
Friends	97	87.4
Doctors	29	26.1
Internet	37	33.3
Pharmacy	54	48.6
Street vendors	1	0.9
From clinics without consulting the doctor	14	12.6
Others	3	2.7
Where do you get the hormones now? (multiple choice)		
Party/gatherings	1	0.9
Prescription from my regular doctor	9	8.1
Internet	29	26.1
From friends and partner	30	27.0
From street vendors	1	0.9
From pharmacy without prescription	79	71.2
From traditional drug store	17	15.3
From clinic without doctor's consultation	18	16.2
Others	3	2.7
Are you on any estrogen?		
Yes	111	78.7
No	30	21.3
No. of estrogens		
1	59	53.2
2	28	25.2
3	24	21.6
Total estrogen dosage (pg/dL)	0.73 (0.03–10)	
Estrogen dose (pg/dL)		
<4	72	64.9
≥4	39	35.1
Are you taking any antiandrogens?		
Yes	55	49.5
No	56	50.5
No. of antiandrogens		
1	42	76.4
2	13	23.6
Medroxyprogesterone	15	27.3
Cytoperone	19	34.6
Progesterone	34	61.8
How are you taking the hormones currently” (multiple choice)		
Oral	81	73.0
Patch	15	13.5
Injection	61	55.0
Others	2	1.8
Had physical examination or blood test before commencing the hormonal therapy		
Yes	14	12.6
No	97	87.4
Other medications		
Insulin	2	1.8
Antihyperglycemics	2	1.8
Antidepressants	2	1.8
Steroids	3	2.7
Antiretroviral	3	2.7
Cost of hormones per month	M=141.51, Med=100 (0–1000)	
Affordability		
Very bad	2	1.8
Bad	4	3.6
Good	82	73.9
Very good	16	14.4
Excellent	7	6.3
Satisfied with the current hormone		
Very bad	1	0.9
Bad	6	5.4
Good	72	64.9
Very good	18	16.2
Excellent	14	12.6
Complications		
Yes	31	27.9
No	80	72.1
Any complications past year?		
Deep vein thrombosis	4	3.6
Hypercholesterolemia	20	18.0
Liver complications	2	1.8
Headaches/migraines	54	48.6

Qualitative interviews also pointed toward pharmacy, friends, and the internet. Hormones were used by trial and error, according to how they felt their body responded. Taking higher doses to quicken transition was quite common.

“I have been taking hormones since I was 14 years of old. I started taking hormones after looking at my friend, my senior (trans woman), her skin was so beautiful … with breasts. So, I asked her, she said she took hormones. She taught me how to take hormones. I was naïve then, I thought there were no female hormones in the body, so I took 4 tablets a day, instead of the 1 recommended by her, I felt dizzy and hot so I stopped and only at the age of 17 did I start again … it was friends who recommended but I have gone to a pharmacy and consulted a pharmacist who recommended a different hormone … Now I get my supply from pharmacy and friends … sometimes from Thailand …” (Respondent 5)

“Dr Google (laughs), that is my main source of information … but I took too many hormones … many side effects … I have met an NGO doctor who advised me about hormones … but only once” (Respondent 3)

“… pharmacy … I have also bought from Thailand, injectables … I have gone with friends and sometimes alone to buy. We will buy a lot each time we go. Now, I am on oral hormone after seeking advice from my friend, she was advised by a doctor to take this hormone” (Respondent 4)

All were on estrogens, most (53.2%) on a single type with a mean dose of 0.73 mg (range 0.03–10 mg); however, majority (64.9%) were on <4 mg. Only about half (49.5%) were on progesterone, most (76.4%) on a single type; 62.8% were on progesterone, 34.6% on cytoperone, and 27.3% on medroxyprogesterone. Oral (73.0%) and injections (55.0%) were the common manner of administration. Most (87.4%) did not undergo a physical or blood test before commencing.

In-depth interviews showed no physical examination or blood test was conducted before and when on hormones because they felt there were no trained doctors to counsel them.

“… no, never did a blood test (to check hormone levels). I have tried consulting a doctor in a private hospital, but he said he only has experience dealing on hormone matters concerning women only … he has no experience dealing with trans. The problem here (in Malaysia) is that we don't have experts to offer advice on hormone use. I hope the policy makers appoint a doctor who knows about trans women. There used to be a doctor in XX hospital (public hospital) but he wasn't an expert, but we could consult him. He would offer hormones … but now he is transferred … There are doctors who are trained on transgender issues but only limited to HIV and STI, not concerning hormones” (Respondent 3)

The mean cost of the hormonal therapy was RM141.51 per month, with most rating the affordability (73.9%) and satisfaction (64.9%) as good. Although most who were interviewed concurred, but some did lament stating unemployment and economic burden.

“for me I feel it is expensive … some hormones in Malaysia are quite pricey. I think for someone who first starts to transition and wants to start hormones, it is costly, especially if not working … if employed they can still afford it…for me I think it is still okay” (Respondent 4)

“the cost is not bad … sometimes I buy online. Well, even if its three or four hundred (will buy) … want to look beautiful … the heart wants” (Respondent 6)

Well, if it (hormones) works, (I am) satisfied. If it doesn't, (I) buy a different kind, keep changing until I find one that suits me” (Respondent 12)

Most (72.1%) did not report complications and those that did, mostly complained of headaches/migraines (48.6%).

During the interviews, no serious complication was mentioned.

“side effects? no, so far I don't have any … well, maybe increase in body weight, some people who take hormones don't have this (increase in weight), some do … my weight increased a lot, about 100kg plus with hormones. Even when I stop, the weight doesn't decrease, just maintains” (Respondent 5)

“… tired … sometimes when I take the hormones, I have difficulty sleeping, body becomes hot, too hot that I can't sleep. That is why sometimes I take and sometimes I don't” (Respondent 12)

Among those taking hormones, the mean total testosterone and estradiol levels were 496.3 ng/dL (8.65–2721.33) and 21,355.88 pg/dL (1000–100,000), respectively. Only 41.4% had adequate testosterone and only 3.6% had adequate estradiol blood levels. As shown in Table 2, except for total cholesterol (53.2%) and LDL (64.0%), all other blood results were within normal range.

**Table 2.** Blood Test Results

Variable	Normal	Abnormal
Urea		
Normal	111	100
Abnormal	0	0.0
Creatinine		
Normal	99	89.2
Abnormal	12	10.8
Potassium		
Normal	109	98.2
Abnormal	2	1.8
Total cholesterol		
Normal	52	46.8
Abnormal	59	53.2
Triglycerides		
Normal	74	66.7
Abnormal	37	33.3
Low-density lipoprotein		
Normal	40	36.0
Abnormal	71	64.0
High-density lipoprotein		
Normal	106	95.5
Abnormal	5	4.5
Total bilirubin		
Normal	109	98.2
Abnormal	2	1.8
Alkaline phosphatase		
Normal	107	96.4
Abnormal	4	3.6
SGPT		
Normal	105	94.6
Abnormal	6	5.4
SGOT		
Normal	106	95.5
Abnormal	5	5
Testosterone	496.3 (8.65–2721.33)	
Adequate	146	41.4
Inadequate	65	58.6
Total estradiol	118.04 (10–1000)	
Inadequate	111	100

SGOT, serum glutamic oxaloacetic transaminase; SGPT, serum glutamic pyruvic transaminase.

**FIG. 2.**
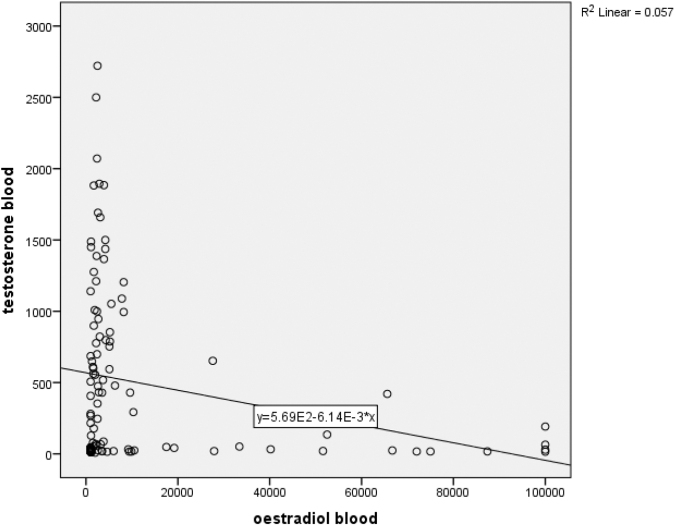
Correlation between blood testosterone and blood estrogen.

Table 3 and Figure 2 show that there is a statistically significant correlation between blood testosterone and blood estrogen (*R*^2^ 0.012. B−9.273 [95% confidence interval −16.44 to −2.11], *p*=0.012). There is no significant correlation between blood estrogen and estrogen dose, medroxyprogesterone, cypeterone, and progesterone.

**Table 3.** Factors Related to Blood Estrogen

Dependent variable	*n*	Mean (SD)	*R* ^2^	Coefficient (95% CI)	*p*
Blood testosterone	111	496.29	0.057	−9.273 (−16.44 to −2.11)	0.012
Estrogen dose	111	0.73 (1.35)	0.004	1062.42 (−2305.76 to 4429.61)	0.533
Medroxyprogesterone	15	150 (0)	—	—	—
Cypeterone	19	50 (0)	—	—	—
Progesterone	34	51.47 (8.58)	0.007	−316.42 (−1690.15 to 1057.30)	0.642

CI, confidence interval; SD, standard deviation.

As shown in Table 4, there is no statistically significant correlation between blood testosterone and medroxyprogesterone, cypeterone, progesterone, and estrogen dose.

**Table 4.** Factors Related to Blood Testosterone

Dependent variable	*n*	Mean (SD)	*R* ^2^	Coefficient (95% CI)	*p*
Medroxyprogesterone	15	150 (0)	—	—	—
Cypeterone	19	50 (0)	—	—	—
Progesterone	34	51.47 (8.58)	0.016	−7.26 (−27.60 to 13.09)	0.473
Estrogen dose	111	0.73 (1.35)	0.0003	−7.59 (−94.38 to 79.19)	0.863

## Discussion

The demographics of the participants differed from the Malaysian population, where median income is reported as RM5873 and unemployment rate of 2.9%. The age group is within the majority Malaysian population of between 16 and 64 years.^27^

The prevalence of hormone use in this study is about the same as another study (88.6%) conducted in central Malaysia^24^ and in Thailand (88.6%).^28^ However, these rates are higher than those reported in the United States, 30.3–68.7%^22,29^ and Canada, 43.0%.^23^ Just like the study in central Malaysia^24^ the hormones were procured, without first consulting a health care provider, from local pharmacies and friends.^24^ This nonprescription use of hormones is higher than the 26.8% reported in Ontario, Canada,^23^ 49.1% in San Francisco, and 23% in New York, United States,^22,29^ where hormones are only available by prescription as compared with in developing countries.^10,23^

Costs and wanting to accelerate the transition process are reasons for nonprescription hormone use, and the lack of access to trans-friendly clinics^9,23,25^ could be a reason for the nonofficial source of information.^24^ In the United States, 73% listed a physician as the source of information on hormone regimens.^25^ Because of the laws that discriminate and discourage transition process, the participants in this study did not first consult nor regularly followed up with a doctor for their hormone use.

In contrast, because of the seriousness of the transition process, in most developed countries a complete checkup before beginning the transition process^1,3,8,9,26^ is common. However, even in the west, not all health care providers are equipped with sufficient training^3^ and in the case of United Kingdom, the waiting list is long.^16^ In the United States, only 58% completed a medical evaluation before starting hormone treatment.^25^

The two main medications used by trans women are estrogen therapy and androgen-lowering therapies.^1,24,25^ In Malaysia^24^ and in parts of Middle East, because of the poor availability of estrogens, combined oral contraceptive products are used,^16^ which may result in either inadequate or excess use. Similar to the literature review by Moore et al.,^10^ oral delivery was the most common method of hormone use in this study, which contrasts to a study in central Malaysia where injectables were more common.^24^

Most of the participants in this study did not report any major adverse effects. This is not surprising as there is only a moderate risk to hormone therapy^13^ although when initiated and monitored under supervision of a medical professional.^10^ Blood tests in this study showed abnormal total cholesterol and LDL results. Two systematic reviews commissioned to support guidelines to develop clinical practice guidelines found significantly higher serum triglycerides^3^. Similar finding was shown in a meta-analysis.^30^ But not all studies reported increase in lipid parameters^15,31^ although other common side effects were reported,^9,24,25,32^ which was not found in this study.

Because of unsupervised hormone use, this study showed inadequate testosterone and estradiol blood levels. This is in contrast to studies in the United States.^2,15^ However, it is important to remember that there is a wide individual variability in the amount required. Studies in the United States found even with 4 mg daily^14^ and self-administered high-dose regimens did not lead to dramatic clinical changes.^10^

Similar to the finding of this study, a study in the United States found the measured serum estrogen levels did not correlate with dosage of estrogen administered,^12^ but there are studies that found a significant correlation.^2,14^ Initiation of estrogen therapy should lower testosterone levels by way of negative feedback of the hypothalamic–pituitary–gonadal axis^33^ as was found in this study, but in contrast no such correlation was found in studies in the United States.^12,14^

## Conclusion and Limitations

This mixed method study provides important information concerning hormone intake among trans women in Malaysia. Because there is no avenue for trans women to seek advice, there is concern of the health risks related to self-prescribed hormone therapy. Primary health care doctors in Malaysia should be equipped with the knowledge of hormone use in the trans community, they should be able to identify gender dysphoria and importantly communicate sound advice in relation to hormone use.

Considering the sensitive nature of the study, snow ball sampling method was the best available option, but this sampling method is not representative of trans women population in Malaysia. Although the research assistant was available for clarification, participant not understanding the questions is a concern. Another limitation is that the conjugated estrogens taken by the participants in this study may not be accurately measured by estradiol essays.

## Abbreviations Used

CI: confidence interval
LDL: low-density lipoproteins
NGO: nongovernmental organization
SD: standard deviation
SGOT: serum glutamic oxaloacetic transaminase
SGPT: serum glutamic pyruvic transaminase
